# Intermittent theta burst stimulation in adolescents and young adults with depressive disorders: protocol of a randomized, sham-controlled study with a sequential Bayesian design for adaptive trials

**DOI:** 10.1007/s00406-024-01926-5

**Published:** 2024-10-10

**Authors:** Gerrit Burkhardt, Simon E. Blackwell, Miaoxi Chen, Lisa Feldmann, Jonas Björklund, Esther Dechantsreiter, Lucia Bulubas, Stephan Goerigk, Daniel Keeser, Peter Falkai, Ellen Greimel, Peter Bechmann, Gerd Schulte-Körne, Alkomiet Hasan, Wolfgang Strube, Frank Padberg

**Affiliations:** 1 Department of Psychiatry and Psychotherapy, LMU University Hospital, Munich, Germany; 2 Department of Clinical Psychology and Experimental Psychopathology, Institute of Psychology, Georg-August-University of Göttingen, Göttingen, Germany; 3 Dept. of Child and Adolescent Psychiatry, Psychosomatics and Psychotherapy, LMU University Hospital, Munich, Germany; 4 Department of Psychiatry, Psychotherapy and Psychosomatics, Faculty of Medicine, University of Augsburg, Augsburg, Germany; 5 Charlotte Fresenius Hochschule, University of Psychology, Munich, Germany; 6 German Center for Mental Health (DZPG), Partner Site Munich-Augsburg, Munich, Germany

**Keywords:** Major depressive disorder, Depression, Transcranial magnetic stimulation, Theta burst stimulation, Adolescents, Adaptive trial design

## Abstract

Intermittent theta burst stimulation (iTBS), a variant of repetitive transcranial magnetic stimulation (rTMS), is an established treatment for adults with major depressive disorder (MDD). Due to its favorable safety profile, iTBS is also a promising early intervention in the transition phase from adolescence to early adulthood, but this has not been systematically investigated to date. Thus, the EARLY-BURST trial investigates the efficacy and safety of iTBS over the left dorsolateral prefrontal cortex (lDLPFC) in treatment-seeking young patients (age 16–26 years) with depressive disorders (i.e. major depressive disorder, persistent depressive disorder, bipolar depression), allowing for relevant co-morbidities. Participants have not received antidepressant or antipsychotic medication during the last 12 months except for short-term (< 2 weeks) on-demand medication. The trial will employ a novel sequential Bayesian, randomized, double-blind, parallel-group, sham-controlled design. Up to 90 patients at two clinical sites (Munich, Augsburg) will be randomized 1:1 to the treatment groups, with sequential analyses starting after 26 patients in each group completed the treatment. The primary outcome will be the difference in depression severity at week 6 (post-treatment visit) between active iTBS and sham iTBS, assessed with the Montgomery-Åsberg Depression Rating Scale (MADRS). The trial is planned to be expanded towards a three-arm leapfrog design, contingent on securing additional funding. Thus, in addition to potentially providing evidence of iTBS’s efficacy in adolescents and young adults with depressive disorders, the EARLY-BURST trial aims at setting the stage for subsequent platform trials in this dynamic research field, where novel adaptive study designs are required to meet the need for rapidly testing promising new vs established rTMS protocols.

*Trial registration:* DRKS00033313.

## Background

Depressive disorders emerge with a first peak age of onset of 20.5 years [1] and are a major contributor to the global burden of disease among adolescents and young adults [2]. Current neurobiological treatments for this demographic are often adapted from adult treatments with minimal adjustments and have limited supporting evidence [3]. Consequently, prioritizing the development of early interventions for depression has been recognized as crucial by patients, informal carers, and clinicians (https://www.jla.nihr.ac.uk/priority-setting-partnerships/depression/).

Repetitive transcranial magnetic stimulation (rTMS) of the dorsolateral prefrontal cortex (DLPFC) is an effective, well-tolerated treatment option for adults with major depressive disorder (MDD) who have not sufficiently benefitted from antidepressant pharmacotherapy, as demonstrated by multicenter trials [4–6]. A recent meta-analysis supports the notion that rTMS of the DLPFC might even qualify as an antidepressant therapy across diagnostic categories [7]. In contrast, the application of rTMS for treating depressive disorders in adolescents and young adults has yielded mixed results. A recent meta-analysis encompassing both open-label and randomized controlled trials (RCT) revealed significant but heterogeneous effects of rTMS on depressive symptoms and response rates among young patients [8]. The largest RCT in adolescents to date even failed to show superior effects of a conventional 10 Hz rTMS protocol compared to sham in patients with non-response to 1–4 adequate pharmacological trials [9]. Conversely, a smaller RCT employing neuronavigation-guided, high-frequency rTMS reported significant short-term amelioration of suicidal ideation in treatment-naive patients with MDD [10]. Furthermore, an open-label trial suggested that intermittent theta burst stimulation (iTBS), a newer and shorter rTMS protocol established in adults [5, 11], might be a feasible and effective treatment option for adolescents [12], although a subsequent RCT comparing once and twice-daily TBS with sham TBS as an add-on to antidepressant medication showed no significant benefits over a short treatment period of 10 sessions in two weeks only [13]. To our knowledge, there is a critical lack of RCTs investigating the efficacy of iTBS for depressive symptoms in adolescents and young adults without concomitant pharmacotherapy. Moreover, studies have not assessed long-term treatment effects over several months. Given the broad spectrum of potential iTBS settings and the ongoing neurodevelopmental processes in younger individuals–which may alter their treatment response compared to adults [14]–it is likely that parameters such as stimulation dose, treatment target, frequency, duration, and patient selection criteria, need to be systematically refined to achieve optimal results in later confirmatory trials. In traditional research frameworks, such optimizations are typically conducted through multiple pilot trials. This approach often leads to protracted, resource-intensive development processes and a high risk of early false positive findings [15]. For example, recent multicenter RCTs investigating transcranial direct current stimulation (tDCS) for MDD within a narrow set of treatment parameters despite a lack of robust early clinical validation [16–18], have resulted in inconsistent findings and provided limited guidance on how to improve the efficacy of the intervention [19]. In contrast, adaptive trial designs, initially developed in oncology, offer an alternative research strategy. These designs allow for more efficient and systematic testing of promising novel interventions. The use of adaptive designs within mental health has been increasingly advocated to leverage innovation and accelerate treatment development [15, 20].

In the EARLY-BURST pilot trial, we aim to assess the efficacy and safety of a six-week course of iTBS targeting the left DLPFC versus sham iTBS in reducing depressive symptoms in treatment-seeking adolescents and young adults with MDD, persistent depressive disorder (PDD), or bipolar disorder with current depression (BD) who have not received antidepressant or antipsychotic medication in the past year, except for short-term on-demand use (less than 2 weeks). On an exploratory basis, we will compare treatment groups across the domains of self-reported depression, anxiety, stress, anhedonia, functioning, and over a 6 month follow-up period. To optimize the use of research resources, our study will employ a Bayesian sequential analysis design [15, 21], which facilitates the early detection of effective vs ineffective treatment protocols [22]. This approach not only promises to reduce the burden on participants in the event of negative outcomes but, crucially, also enables the trial to be transformed into an adaptive multi-arm design, where protocol refinements can be iteratively tested to further enhance the efficacy of the intervention. We currently seek additional funding for such an extension.

## Methods

### Trial design

**Fig. 1:**
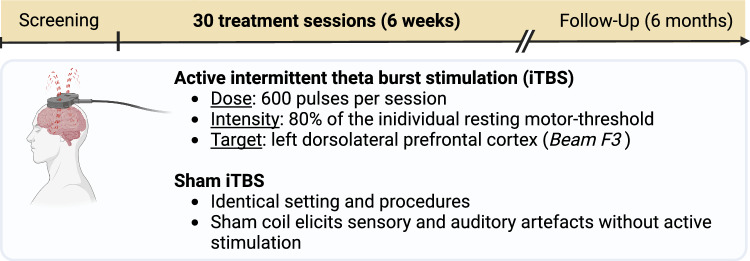
Treatment groups

The EARLY-BURST trial is a sequential Bayesian, randomized, double-blind, parallel-group, sham-controlled pilot trial testing the superiority of six weeks of intermittent theta burst stimulation (iTBS) of the left dorsolateral prefrontal cortex (lDLPFC) versus sham iTBS (see Fig. 1). The primary outcome will be the difference in depression severity at week 6 (post-treatment visit) between active iTBS and sham iTBS, assessed with the Montgomery-Åsberg Depression Rating Scale (MADRS).

**Fig. 2:**
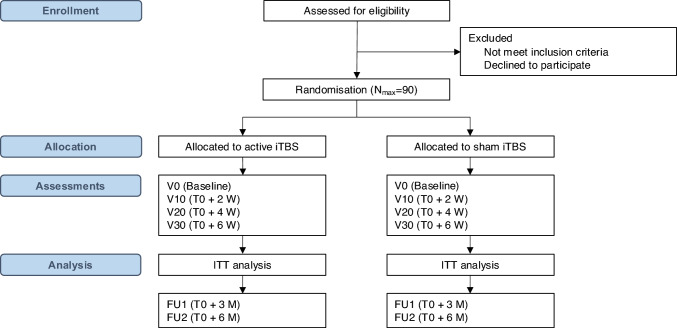
Study flow. *N*_max_ maximum sample size, *iTBS* intermittent theta burst stimulation, *ITT* intention-to-treat, *W* weeks, *V* visit, *M* months, *FU* follow-up

Trial outcomes and exploratory measures will be assessed in person at the participating study centers using the CentraXX patient app (https://www.kairos.de/en/products/centraxx-patient-app/). Study visits are scheduled for screening, at baseline, at two-time points during treatment (week 2 and week 4), and upon completion of the intervention (week 6; see Fig. 2). Follow-up visits will be conducted three and six months after randomization. All patients are required to provide written informed consent prior to participating in any study-related procedures. The trial started recruitment of patients in April 2024 and will continue recruiting until April 2027. The study will be reported in accordance with the Consolidated Standards of Reporting Trials Statement 2010 and the extensions for reporting randomized pilot and feasibility trials [23], for reporting outcomes in clinical trials [24], and the template for intervention description and replication (TIDieR) checklist [25]. This trial protocol follows the SPIRIT guideline [26].

### Study setting

The trial will be conducted in an urban setting in Germany at the Center for Non-invasive Brain Stimulation Munich-Augsburg (CNBS^MA^), a specialized in- and outpatient service at the LMU University Hospital Munich's Departments of Psychiatry and Psychotherapy and Child and Adolescent Psychiatry, Psychosomatics and Psychotherapy and the University of Augsburg's Department of Psychiatry, Psychotherapy, and Psychosomatics.

### Participants

Overall, a maximum of 90 treatment-seeking female, male, and non-binary in-and out-patients with depressive disorders and no antidepressant or antipsychotic medication during the last 12 months, except for short-term (< 2 weeks) on-demand medication, are planned to be recruited. We will allow co-morbidities, except for severe psychopathology, likely to interfere with the study procedures and conditions unlikely to benefit from treatment. Inclusion criteria are (1) age 16–26 years; (2) diagnosis of MDD, PDD, or BD (Diagnostic and Statistical Manual of Mental Disorders, Fifth Edition, [DSM-5] criteria; assessed with the Diagnostic Interview for Mental Disorders [DIPS]); (3) no antidepressant or antipsychotic medication during the last 12 months, except for short-term (< 2 weeks) on-demand medication; (4) fluent in reading and speaking German; (5) capable and willing to provide informed consent (has to be confirmed by an independent physician; in the case of minors, the consent of the legal guardians will be obtained). Exclusion criteria are (1) positive screening for acute mania (defined by reaching the threshold “mild or greater” on at least one question from the Adult DSM-5 Self-Rated Level 1 Cross-Cutting Symptom Measure [from here on referred to as “APA screener”] Mania domain); (2) positive screening for acute psychosis (defined by reaching the threshold “slight or greater” on at least one question from the APA screener Psychosis domain); (3) positive screening for Obsessive–Compulsive Disorder (defined by reaching the threshold “mild or greater” on at least one question from the APA screener Repetitive Thoughts and Behaviors domain); (4) severe borderline typical psychopathology, defined as a very-high mean score on the self-reported Borderline Symptom List (BSL-23) of ≥ 2.67; (5) primary substance use disorder except for nicotine and caffeine (DSM-5, assessed with the DIPS); (6) acute risk for suicidality, assessed by the Columbia Suicide Severity Rating Scale (C-SSRS; patient agrees to item 4 and/or item 5); (7) history of brain surgery, significant and clinically relevant brain malformation or neoplasm, head injury, stroke, dementia or other neurodegenerative disorder; (8) history of seizures; (9) previous brain stimulation treatment (rTMS, transcranial direct current stimulation, electroconvulsive therapy, vagus nerve stimulation, deep brain stimulation); (10) cardiac pacemakers, intracranial implants, or metal in the cranium; (11) antiepileptic drugs and/or benzodiazepines corresponding to > 1 mg lorazepam/day; (12) severe somatic comorbidity as judged by the study physician; (13) pregnancy (negative urine HCG test in women); (14) any clinically relevant findings in a structural MRI safety check (evaluated by a neuroradiologist); (15) investigators, site personnel directly affiliated with this study, and their immediate families (immediate family is defined as a spouse, parent, child or sibling, whether by birth or legal adoption). Participants will be recruited after referral or personal inquiry at the CNBS^MA^ using offline and online materials approved by the institutional ethics committee.

### Randomization, assignment concealment, and blinding

Eligible patients will be randomized 1:1 to the active iTBS or sham iTBS treatment arms. An independent statistician (SEB) will do the randomization, using an in-house R script (with random sequences from random.org) to generate randomly permuted blocks of varying lengths, stratified by study sites (LMU University Hospital vs. University of Augsburg) and biological sex at birth. After baseline assessments, clinical raters will provide participant stratification information to SEB. SEB will then send one of two non-descriptive, alphanumeric codes via mail to the TMS operators, matching markings on either the active iTBS or sham iTBS coil. The markings will be applied by an independent clinician not involved in the administration, recruitment, treatment, medical oversight, analysis, or any other study-related procedure prior to the study initiation. Apart from the non-descriptive markings, the sham coils look identical to the active stimulation coils, generate identical sounds, and have similar weights. Furthermore, they emit minimal magnetic field strengths with which only the nearest area (such as the scalp) is stimulated, producing a twitching sensation without neural activity. Thus, operators will be able to select the correct coil while remaining blinded to the treatment condition. Study raters will not be involved in the treatment sessions and thus will be kept blinded. Treatment conditions will be revealed after the final study analysis has been conducted.

### Intervention

The treatment will encompass 30 sessions of either lDLPFC iTBS or sham iTBS over 6 weeks (5 sessions per week, daily from Monday to Friday). All treatment sessions will be performed in person at the respective treatment sites by assistant medical technicians, post-graduate psychologists, research assistants, and medical students under the supervision of a medical doctor. All TMS operators will receive sufficient on-site training for conducting the treatment. During the session, patients will be seated in comfortable chairs in a neutral-colored room. No other patients or personnel will be present except for the operators, who will be instructed not to converse with the participant during stimulation. Patients will wear hearing protection during stimulation.

Active iTBS treatment will follow the original protocol by Huang et al. [27] except for using 80% resting motor threshold (rMT) instead of active motor threshold (aMT), but also comprising bursts of 3 pulses at 50 Hz repeated at 200 ms intervals (5 Hz), with an intertrain interval of 8 s, for a total of 600 stimuli (i.e. lasting about 3 min). In a recent study, we characterized the neural response to this iTBS protocol applied over the left DLPFC using interleaved TMS-fMRI [28]. The individual rMT will be determined as the lowest stimulation intensity that elicits motor-evoked potentials (MEPs) with a minimum amplitude of 0.050 mV from the relaxed abductor pollicis brevis muscles in at least 5 out of 10 stimulations of the left motor hotspot region (M1 hand). The individual lDLPFC will be located according to the established Beam F3 method [29]. Motor threshold determination and active and sham iTBS will be performed using the PowerMag Clinical 100 stimulator (Mag & More GmbH, Munich, Germany) with a marked “PMD70-PCOOL” for threshold determination, a separate, unmarked “PMD70-PCOOL” for active iTBS, and an unmarked “PMD70-PCOOL-SHAM” coil for sham iTBS. The manufacturers were not involved in the study design and will not be involved in collecting data, analyzing data, interpreting data, or writing the report. Modifications to the outlined treatment protocol will not be allowed.

During the 6 week treatment phase, concurrent standard psychotherapeutic and psychosocial interventions will be allowed, but psychoactive pharmacotherapy, except for pre-defined on-demand medication, will not be allowed. On-demand medication will include zopiclone (up to 7.5 mg/day orally), benzodiazepines up to a dose equivalent to Lorazepam 1.0 mg/day orally, quetiapine up to a dose of 50 mg/day orally, promethazine up to a dose of 50 mg/day orally, as well as ibuprofen, Paracetamol, ASA for treatment of local pain, dental pain or headaches, as necessary. During follow-up, we will observe patients under naturalistic conditions without interfering with their treatment.

## Measures

Table 1 presents an overview of measures that are assessed in this trial.

**Table 1:** Schedule of visits

Visit	Pre-treatment		Treatment			Follow-up	
	Screen	Baseline	V10	V20	V30	FU1	FU2
Week	− 2 to 0		2	4	6	12	24
Screening assessments							
DIPS	x						
Medical and psychiatric history	x						
Sociodemographic information	x						
APA screener	x				x	x	x
BSL-23	x				x	x	x
Outcome assessments							
MADRS		x	x	x	x	x	x
DASS-21		x	x	x	x	x	x
DARS		x	x	x	x	x	x
WHO-DAS 2.0		x			x	x	x
Exploratory assessments							
ISI		x			x	x	x
CTQ		x			x	x	x
PID5BF + M		x			x	x	x
SNI		x			x	x	x
UCLA		x			x	x	x
RSQ		x			x	x	x
Safety assessments							
C-SSRS	x	x	x	x	x		
Physical exam	x				x		
Vital signs	x				x		
Urine HCG*	x						
Treatment-related assessments							
Blinding check			x	x	x		
CEQ		x	x	x	x		
Optional assessments							
MRI		x			x	x	

*Women only

DIPS, Diagnostisches Interview bei psychischen Störungen; APA screener, DSM-5 self-rated level 1 cross-cutting symptom measure—adult version; BSL-23, Borderline Symptom List, 23-item version; MADRS, Montgomery-Åsberg Depression Rating Scale; DASS-21, Depression, Anxiety, and Stress Scale, 21-item version; DARS, Dimensional Anhedonia Rating Scale; WHO-DAS 2.0. World Health Organisation Disability Assessment Schedule (WHODAS 2.0) self-report version; ISI, Insomnia Severity Index; CTQ, Childhood Trauma Questionnaire; PID5BF + M, Persönlichkeitsinventar für DSM-5 und ICD-11—Kurzform Modifiziert; SNI, Social Network Index; UCLA, UCLA Loneliness Scale; RSQ, Rejection Sensitivity Questionnaire; C-SSRS, Columbia-Suicide Severity Rating Scale; CEQ, Credibility/Expectancy Questionnaire; MRI, Magnetic resonance imaging

### Primary outcome

The primary outcome will be the difference in MADRS scores at week 6 (post-treatment visit) between active iTBS and sham iTBS, controlling for baseline MADRS scores. The MADRS is a clinician-rated measure of depression severity that assesses ten common depressive symptoms (apparent sadness, reported sadness, inner tension, reduced sleep, reduced appetite, concentration difficulties, lassitude, inability to feel, pessimistic thoughts, and suicidal thoughts) on a scale from 0–6. The overall score ranges from 0 to 60, with higher MADRS scores indicating more severe depression. We will use the structured interview guide for the MADRS (SIGMA)[30] to improve the reliability of assessments between study raters. All raters will receive video-based SIGMA training, during which they will rate two example video interviews.

### Secondary, exploratory outcomes

Secondary outcomes will explore group differences in (1) MADRS score changes from baseline to 3 and 6 months; (2) response rates (≥ 50% MADRS score reduction) at week 6, and 3 and 6 months; (3) remission rates (MADRS score ≤ 10) at week 6, and 3 and 6 months; (4) changes in self-reported depression, anxiety, and stress using the Depression, Anxiety, and Stress Scale, 21-item version (DASS-21)[31] at week 6, and 3 and 6 months; (5) the change of self-reported anhedonia using the Dimensional Anhedonia Rating Scale (DARS) [32] at week 6, and 3 and 6 months; and (6) change of self-reported health-related functioning using the World Health Organisation Disability Assessment Schedule (WHODAS 2.0) [33] at week 6, and 3 and 6 months.

### Safety and tolerability outcomes

Safety outcomes will be the group differences in (1) rates of reported adverse events (AEs); (2) change of vital signs (blood pressure and heart rate) and Body Mass Index (BMI); and (3) rates of reported instances of suicidality; all measured at week 6 (post-treatment visit). Suicidality will be assessed at every visit using the Columbia-Suicide Severity Rating Scale (C-SSRS) [34], an interviewer-rated scale that measures the current intensity of patients’ specific suicidal ideation on the day of assessment. A patient will be identified as experiencing acute suicidality if they affirm item 4, item 5, or both. Upon such identification, treatment will be halted immediately, and the patient will be directed to suitable crisis intervention services for urgent care. Tolerability outcomes will be the group differences in (1) rates of participants who discontinue the treatment; and (2) rates of participants who discontinue the treatment due to an AE.

### Other assessments

The following variables and measures will be collected at baseline and/or during the course of the trial as potential moderators and mediators of treatment: Demographic and clinical variables (e.g., age and gender, prior psychiatric treatment, medical co-morbidities); self-reported symptoms of insomnia using the Insomnia severity index (ISI) [35]; self-reported experiences of adverse childhood experiences using the Childhood Trauma Questionnaire (CTQ) [36]; self-reported maladaptive personality traits, using the PID5BF + M, a validated, expanded German version of the Personality Inventory for DSM-5 (PID-5) [37]; self-reported participation in social relationships using the Social Network Index (SNI) [38]; self-reported loneliness using the UCLA Loneliness Scale (UCLA) [39]; rejection sensitivity using the Rejection Sensitivity Questionnaire (RSQ) [40]; and outcome expectation beliefs using the Credibility/Expectancy Questionnaire (CEQ) [41]. We will explore participants' beliefs about their intervention allocation by asking them to guess their assigned treatment condition, rate their confidence in this guess, and provide potential reasons for their guess at weeks 2, 4, and 6 (post-treatment) using a custom-designed questionnaire.

Furthermore, participants will be asked to participate in optional, exploratory MRI assessments prior to treatment (mri_t0; additional MRI sequences will be added to the mandatory safety measurement), within two weeks after the end of treatment (mri_t1), and 3 months after randomization (mri_t2). MRI data will be used to explore potential longitudinal effects of iTBS on brain anatomy and functioning, identify potential moderators of treatment response, and investigate dose–response relationships using electric-field modeling. Imaging sequences will encompass anatomical (such as T1-weighted and T2-weighted structural sequences, diffusion tensor imaging, DTI) and functional scans during resting state (rsfMRI). Participants will receive a reimbursement of 50 € per MRI assessment.

### Statistical methods and analysis

This protocol pre-specifies only the primary sequential analyses; others are exploratory. Safety assessments will be descriptive. All analyses will be conducted in R [42]. The analysis scripts are available at 10.17605/OSF.IO/B397U.

**Fig. 3:**
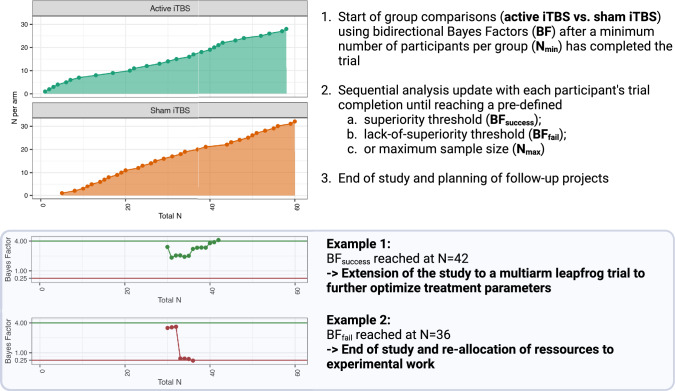
Sequential Bayesian analysis design

Following the methodology of Blackwell et al. [15], we will employ sequential analyses (see Fig. 3) with directional Bayes factors (BFs) to assess the efficacy of active and sham iTBS on the primary outcome. BFs [43] essentially calculate the probability of the observed data under the alternative hypothesis (active iTBS reduces MADRS scores more than sham) divided by the probability of the observed data under the null hypothesis (no superiority of active iTBS). The progression of the trial depends on pre-defined parameters: the minimal sample size per arm, N_min_, at which sequential analyses are initiated; a maximum sample size, N_max_; a BF threshold indicating lack of superiority, BF_fail_; and a BF threshold indicating superiority, BF_success_. The specific parameter set was determined by simulation (see below). The trial will halt upon reaching any threshold or N_max_. In the latter case, discussions on extending recruitment may occur with the funder, pending additional ethical approval. Participants ongoing in treatment at trial cessation will complete it, with their data included in final analyses but not in further sequential analyses.

Analyses will follow an intention-to-treat approach, including all participants randomized to a condition, irrespective of intervention completion or outcome data availability. For sensitivity analyses, participants will be classified as per protocol if they complete the 6-week treatment period and receive at least 20 treatment sessions within six weeks. Sequential analyses will commence once each arm meets the predefined minimum sample size N_min_. Analyses will be updated with each participant’s trial completion. Following Blackwell et al. (2022), missing data will be handled using constrained longitudinal data analysis (cLDA) via the R package nlme [44]. Bayes factors will be estimated with the R package BayesFactor [45] using the t-statistic for the Time x Group interaction and directional default Cauchy prior (rscale parameter = √2/2). Effect sizes (adjusted Cohen's d) and estimated means for the primary outcome, along with their 95% confidence intervals (CIs), will be derived from the cLDA model using the R packages effectsize [46] and emmeans [47]. Analyses will be performed by a statistician (SEB) aware of participant allocation (given the directional nature of the analyses), sharing results with the research team only if a BF boundary is met or after N_max_ is reached.

### Sample size calculation

Using pairwise comparison simulations, we set analysis parameters to achieve a false-positive rate of < 5% and a power of ~ 80% for detecting an effect size of d = 0.6. The choice of effect size was revised after the trial commenced but prior to the start of sequential analysis, based on a recent meta-analysis of iTBS [48]. Initially, we selected an effect-size of 0.8 due to practical considerations, including the absence of specific evidence for iTBS in younger populations and constraints on trial funding. For N_min_ = 26; N_max_ = 45; BF_fail_ = 1/5; and BF_success_ = 5, simulations with up to 15% missing data suggest that these parameters provide a < 5% false-positive rate and 71.35% power to reach the BF_fail_ threshold before recruitment of N_max_ when d = 0; and 81% power to hit BF_success_ before N_max_ for an effect size of d = 0.6(see Table 2). For practical purposes, N_max_ will be operationalized on the basis of the total sample size, that is, when the total number of participants randomized is 2 × N_max_ (i.e., *N* = 90), regardless of the distribution across arms. Simulation scripts and results of the initial and revised sample size calculations are available at 10.17605/OSF.IO/B397U.

**Table 2:** Pairwise comparison simulations using cLDA

	Probability of hitting BF threshold at each participant number (per group)									
	Hit BFfail					Hit BFsuccess				
d	26	30	35	40	45	26	30	35	40	45
− 0.2	73.85	82.45	87.20	90.90	93.40	0.20	0.45	0.45	0.70	0.70
0	46.20	55.60	62.05	67.20	71.35	**1.30**	**1.95**	**2.45**	**2.75**	**3.00**
0.2	18.85	25.15	30.15	33.40	35.40	6.20	9.95	13.10	15.75	17.95
0.3	11.45	15.90	18.65	20.40	22.10	12.85	18.05	22.25	26.50	29.55
0.4	5.45	7.25	8.55	9.70	10.15	21.85	31.10	37.55	42.75	47.45
0.51	2.00	2.95	3.40	3.85	4.00	34.65	45.60	54.10	60.25	66.55
0.6	1.25	1.55	1.85	1.95	2.00	**48.95**	**60.25**	**69.45**	**76.25**	**81.15**
0.71	0.30	0.50	0.55	0.55	0.55	63.10	74.65	82.55	87.15	91.10
0.8	0.05	0.20	0.20	0.20	0.20	74.15	83.35	89.80	92.65	95.35

Bold values indicate false-positive rate and power estimates for an effect size of 0.6

Simulations for the following parameters: N_min_ = 26, N_max_ = 45, BF_fail_ = 1/5, BF_success_ = 5; 15% missing data. Simulations were conducted in RStudio running R version 4.2.2, using 2000 simulations. Estimated effect sizes (d) are a form of Cohen’s d estimated from a t-test on the change scores between groups, with positive d values indicating superiority of the treatment condition to the sham condition. Although d values at intervals of 0.1 were simulated (e.g. 0.2, 0.3, 0.4), the table presents the mean observed effect size across the simulations, which often deviate slightly from these values.

### Patient and public involvement

A patient and public representative (PB) has reviewed the patient information and consent documents, contributed to this manuscript, and will participate in recruitment, safety monitoring, interpretation of results, dissemination of results, and writing of the final report.

### Ethical approval and dissemination

The Ethics Committee of the Medical Faculty of the Ludwig Maximilian University Munich approved the first version of the study protocol (version 1.0) on 09.06.2023 and the latest version (version 1.2) prior to the start of recruitment on 22.03.2024. An amendment of the protocol containing revised sample size calculations is currently prepared for submission to the Ethics Committee. The trial has been registered at the German Clinical Trials Register (https://www.drks.de/drks_web/; DRKS00033313). It will be conducted in accordance with the Declaration of Helsinki and GCP-ICH guidelines. The study's results will be published regardless of the outcome.

### Trial status

Recruitment of participants started on 01.04.2024. As of the submission date of this manuscript, one participant has been enrolled in the trial.

## Discussion

Depressive disorders frequently emerge during the transition phase from adolescence to adulthood, carrying significant adverse health consequences. As shown by prior RCTs in MDD, iTBS targeting the DLPFC is a safe and effective non-invasive brain stimulation (NIBS) intervention that could broaden the range of available treatment options in this transition phase. The EARLY-BURST pilot trial investigates the efficacy and safety of iTBS vs sham iTBS in reducing depressive symptoms in adolescents and young adults. While the use of concurrent pharmacotherapy will be limited to on-demand medication, psychotherapy and other psychosocial interventions will be allowed throughout the trial. Thus, iTBS will be evaluated as an additional treatment in a standard-of-care setting.

Acknowledging the heterogenous and evolving clinical manifestation in young patients [49] and based on meta-analytic evidence for the efficacy of left DLPFC rTMS in depressive syndromes across a transdiagnostic spectrum [7], our study includes adolescents and young adults with MDD, PDD, and BD and allows for pertinent comorbidities, except where such conditions likely disrupt trial procedures (e.g., mania, severe borderline psychopathology) or where alternative treatments are required (e.g., obsessive–compulsive disorder). In contrast to RCTs in adults that advocate for accelerated or high-intensity iTBS protocols to mitigate depressive symptoms [50], we opted for a standard iTBS variant only minimally modified from the original Huang et al. [27] protocol and will apply this protocol for 30 sessions within six weeks. This choice is based on the efficacy of a longer iTBS treatment duration in adults demonstrated in multicenter trials [5], recent trials indicating limited efficacy of shorter treatment protocols (i.e. only 10 sessions within two weeks) in younger patients [13], and target involvement by this protocol as shown in a recent interleaved TMS-fMRI study [28]. Moving away from the usual heuristic selection of treatment parameters in treatment development research, our trial employs a sequential Bayesian design that can be later expanded into an adaptive multiarm leapfrog trial allowing for ongoing refinements of the treatment protocol. Specifically, if the active iTBS group reaches the superiority threshold (BF_success_), it replaces the sham group as the comparison arm. New treatment arms with novel rTMS targets and protocols could then be added and tested within a running platform trial. These arms can be informed by emerging clinical evidence (e.g., introducing bilateral or accelerated stimulation based on results from ongoing trials [51, 52]), participant feedback (e.g. reducing the number of stimulation sessions to minimize attrition), or secondary analyses of current trial data (e.g., shortening the treatment duration if improvement plateaus are observed in the active iTBS group). Using a leapfrog rolling design, each active comparison arm can subsequently be superseded by new arms that demonstrate superiority, while underperforming arms exit the trial. This frees up resources to either introduce alternative treatment settings or concentrate efforts on existing arms. This iterative process continues until researchers determine that an optimized intervention is ready for large-scale confirmatory trials, or it can be perpetually embedded in routine practice as a platform for continually testing new ideas. Compared to traditional treatment development, which typically involves series of small, sham-controlled studies, this approach reduces required sample sizes, minimizes exposure to sham treatments or ineffective versions of the intervention, and rapidly incorporates new knowledge within an existing trial infrastructure, without the need to setup separate parallel trials. Thus, this pilot trial represents the initial phase in a translational framework aimed at systematically optimizing promising treatment modalities prior to launching more resource-intensive confirmatory multicenter trials.

Our trial has several limitations. First, due to practical and financial constraints, we have set a maximum number of participants, at which point the trial will conclude, potentially without delivering conclusive evidence regarding the efficacy of the active treatment. Should preliminary results show promise, discussions on extending the trial with funders may be feasible [53]. Secondly, the scope of this trial does not yet extend to conducting a full sequential adaptive trial capable of comparing various TBS parameters due to current constraints by limited funding. Thirdly, given the absence of studies specifying an age range where DLPFC connectivity is more susceptible to rTMS, we opted to include patients aged 16–26 years. This decision was based on the availability of a structured diagnostic interview validated for depressive disorders within this age group (DIPS). Fourthly, due to the lack of validated measurement instruments for self-reported anhedonia, depression, and anxiety that cover both adolescent and adult age groups, we chose to include the DASS-21 and DARS, despite their validation being limited to adult populations. Lastly, given its limited scale, this pilot trial will likely not be suited to detect moderating or mediating variables with small effect sizes in post-hoc analyses.

In conclusion, the EARLY-BURST trial aims to provide initial evidence on the efficacy and safety of iTBS for reducing depressive symptoms in adolescents and young adults with depressive disorders. Should the results prove positive, this trial could serve as the basis for subsequent iterative optimizations of the tTBS protocol and be extended to an adaptive platform trial. However, refined protocols would still necessitate validation by confirmatory multicenter RCTs prior to clinical application.
